# Processes of supported promotion of physical activity by health professionals: protocol for mixed-methods evaluation within the PROMOTE-PA hybrid effectiveness-implementation cluster randomised controlled trial

**DOI:** 10.1136/bmjnph-2025-001193

**Published:** 2025-08-24

**Authors:** Belinda Wang, Leanne Hassett, Catherine Sherrington, Abby Haynes, Jennifer Cartwright, Kate Purcell, Roslyn Savage, Anne Tiedemann, Sakina Chagpar, Daniel Cheung, Joanna Diong, Kris Rogers, Georgina Clutterbuck, Ben J Smith, Marina Pinheiro

**Affiliations:** 1 Sydney Local Health District Institute for Musculoskeletal Health, Camperdown, New South Wales, Australia; 2 The University of Sydney School of Public Health, Sydney, New South Wales, Australia; 3 The University of Sydney School of Health Sciences, Sydney, New South Wales, Australia; 4 Western Sydney Local Health District, Sydney, New South Wales, Australia; 5 Sydney Health Partners Implementation Science Academy, Sydney, New South Wales, Australia; 6 The University of Sydney School of Medical Sciences, Sydney, New South Wales, Australia; 7 University of Technology Sydney Faculty of Health, Ultimo, New South Wales, Australia; 8 The University of Queensland School of Health and Rehabilitation Sciences, Saint Lucia, Queensland, Australia

**Keywords:** Physical performance

## Abstract

**ABSTRACT:**

**Introduction:**

Physical inactivity is a pressing global health issue. Health professionals have valuable opportunities to promote physical activity to patients across the lifespan, but they report barriers to providing such guidance. The Promotion of Physical Activity by Health Professionals trial aims to deliver implementation strategies to teams of health professionals (n=30 clusters) to address barriers and leverage facilitators within their clinical context to promote physical activity among their patients (n=720) (individuals aged five or above receiving care in outpatient or private settings). This trial will use a hybrid type 1 effectiveness-implementation design to investigate the effect of this support on moderate-to-vigorous physical activity (MVPA) in patient participants compared with a waitlist control. In addition to determining the effectiveness of this physical activity promotion approach, we will conduct a process evaluation that explores implementation within the trial and seeks to identify mechanisms that help explain the findings.

**Methods:**

Our mixed methods process evaluation will employ measures to address determinants across implementation and intervention delivery, guided by a logic model which articulates how the implementation strategies and intervention are intended to work. This is informed by the UK Medical Research Council’s guidance on process evaluations for complex interventions and McKay’s implementation evaluation roadmap. Data will be collected using surveys and interviews with participating health professionals and patients. Implementation outcomes will include adoption, reach, fidelity and dose. Implementation determinants will include feasibility and acceptability. Quantitative data will be summarised using descriptive statistics and presented using tables and narrative synthesis. Qualitative data will be analysed using a qualitative descriptive approach. Later stage qualitative analysis will incorporate emergent findings from the quantitative analysis to develop a nuanced picture combining narrative accounts with descriptive statistics, exploring how and why implementation support influenced key drivers of behaviour change.

**Discussion:**

Results of this process evaluation will improve understanding of implementation within the study and mechanisms which may impact MVPA among patient participants. This evaluation aims to guide future implementation and scale-up of the implementation strategies and interventions to suit varied clinical contexts and future research.

**Trial registration number:**

Australian New Zealand Clinical Trials Registry (ACTRN12623000920695).

**WHAT IS ALREADY KNOWN ON THIS TOPIC**

- Physical activity has many health benefits and health professionals are well placed to promote physical activity to their patients. However, they face a range of barriers, including limited skills in facilitating behaviour change, a lack of suitable local physical activity opportunities and limited knowledge of and trust in available community-based physical activity programmes.

**WHAT THIS STUDY ADDS**

- By using implementation strategies to support health professionals to incorporate physical activity promotion into daily practice, the Promotion of Physical Activity by Health Professionals trial aims to improve physical activity among children, adults and older adults receiving health services. The planned process evaluation will improve our understanding of the factors that influence physical activity promotion by health professionals and the impact on physical activity levels of health service users.

**HOW THIS STUDY MIGHT AFFECT RESEARCH, PRACTICE OR POLICY**

- This process evaluation will identify elements of the implementation strategies that may need to be adapted for future delivery across different healthcare settings to improve the amount and quality of physical activity promotion by health professionals.

## Introduction

 Physical activity (PA) is associated with reduced mortality and the prevention and management of chronic diseases across the lifespan.^1^ There is strong evidence for the physiological and psychological benefits of PA in children and adolescents, and emerging evidence for wide-ranging benefits among those living with disability.^2 3^ PA also holds important benefits for falls prevention and maintaining independence in older adults.^4 5^ Despite this, physical inactivity remains a pressing global health issue, leading to 5.3 million avoidable deaths annually.^6^ To address this issue, the evaluation and widespread implementation of effective PA interventions are urgently needed.

The WHO recommends that PA promotion is integrated into healthcare settings.^7^ Health professionals have valuable opportunities to promote PA to a large group of people across the lifespan^8^ and have a strong interest in supporting their patients to increase PA participation.^9 10^ Barriers to providing this support include limited skills in behaviour change facilitation, a lack of suitable local PA opportunities and limited knowledge of and trust in available community-based PA programmes.^9 11 12^

Promotion of PA by health professionals requires: (a) PA promotion interventions with proven effectiveness that can be delivered within the context of usual clinical care and (b) implementation strategies, that is, methods or techniques to support implementation, that can be tailored to suit local needs. Promising PA interventions and implementation strategies must be amenable to sustainable scale-up across communities and health systems to address population level health.^13^ Therefore, understanding the factors that influence intervention and implementation effectiveness is needed.^13 14^

Alongside an intervention trial, process evaluation can capture the extent and quality of intervention delivery and identify the underlying mechanisms, implementation and contextual factors that influence trial outcomes.^15^ This provides critical information for interpreting trial outcomes and informing adaptations that may better suit different contexts in future implementation efforts.^15^ This is likely to be particularly useful in large-scale, multisite trials where local tailoring of the intervention and/or implementation strategies is needed.^16^

This paper describes the planned methods for a process evaluation to be conducted alongside the Promotion of Physical Activity by Health Professionals (PROMOTE-PA) trial. PROMOTE-PA is a hybrid type 1 effectiveness-implementation cluster randomised trial investigating the effect of PA promotion by health professionals (the intervention), with implementation support from the research team, on PA levels among patients receiving healthcare.^17^

### Process evaluation aims

The aim of the process evaluation conducted alongside the PROMOTE-PA trial is to support the interpretation of trial outcomes and inform future adaptations to the PA promotion intervention and implementation strategies. This will inform a better understanding of a process for adaptation of the implementation strategies with the goal of widespread implementation and scale-up that suits the context and needs of a range of healthcare settings. The research questions are as follows:

#### Intervention delivery level

1. What are the characteristics of the health professionals who delivered PA promotion?
2. Did implementation support increase PA promotion by health professionals?
3. What factors influenced PA promotion by health professionals?
4. What were the health professionals’ perceptions of the acceptability and feasibility of delivering the intervention?

#### Implementation strategies level

1. What were the different implementation strategies offered to support clinical teams and how were they adapted to suit the needs of each clinical team?
2. What was the level of adoption, engagement with and adherence to implementation strategies delivered to health professionals?
3. What elements of the health professionals’ context influenced the selection and use of implementation strategies?
4. What were the health professionals’ perceptions of the acceptability and feasibility of the implementation strategies and their interactions with the research team?

### PROMOTE-PA trial

The primary aim of PROMOTE-PA is to evaluate the impact of PA promotion by health professionals on the PA levels of people who receive outpatient and community health services. The secondary aim is to observe and gather information on the impact of the implementation strategies used to support the clinical teams by conducting this process evaluation. 30 clinical teams will be randomised to either the early implementation support group (immediate receipt of implementation strategies) or the delayed implementation support group (receipt of implementation strategies following recruitment of their anticipated quota of patients). A detailed trial protocol has been published separately^17^ and is briefly described here. This paper focuses on the process evaluation of the trial.

### Participants

Healthcare teams of clinicians from various disciplines (eg, physiotherapists, geriatricians, rehabilitation physicians, nurses) providing outpatient or community-based services within the included New South Wales (NSW) health districts and specialty network, and private clinics will be included.

Patient participants will be 720 community-dwelling adults or school-aged children/adolescents. Patients will be eligible if they are willing to receive additional support to be more active, and can respond to written or verbal questionnaires in English, Arabic or Vietnamese (commonly understood languages according to our expected patient demographic). Exclusion criteria include having a medical condition precluding PA, progressive neurological disease severely affecting function or other conditions affecting participation, for example, delirium, severe psychiatric disorders.

### Interventions

#### PA promotion intervention within routine care

Health professionals will be encouraged to deliver PA promotion within their usual practice to patients based on the 5As (Assess, Advise, Agree, Assist, Arrange) model of brief counselling (table 1).^18^ All health professionals will be encouraged to assess their patients’ PA levels and provide brief advice about PA.

**Table 1:** 5As model^18^ for the delivery of physical activity promotion

Assess	Assess patients’ physical activity participation and influences on their physical activity participation
Advise	Provide specific advice to patients regarding (a) the benefits of physical activity using motivational interviewing techniques, (b) specific physical activity recommendations and (c) suitable physical activity options
Agree	Collaboratively set goals and develop an action plan with patients
Assist	Collaboratively identify barriers and potential solutions with patients, and set up a self-monitoring strategy
Arrange	Arrange referral to a community physical activity programme and/or the PROMOTE-PA Linkage Programme, discuss social support and arrange follow-up

PROMOTE-PA, Promotion of Physical Activity by Health Professionals.

Clinical teams with limited capacity to deliver PA promotion beyond the ‘Assess’ component will be invited to refer their patients to the PROMOTE-PA Linkage Programme delivered by trial staff. Patients will receive up to two telehealth health coaching sessions with a physiotherapist or exercise physiologist to collaboratively develop a tailored PA plan, including referral to suitable PA opportunities/services.

#### Implementation strategies

Implementation strategies will be offered to each clinical team based on an individualised service mapping process conducted after randomisation. Service mapping will involve the research team working with clinical teams to identify readiness for change, current PA promotion practices and associated barriers and facilitators. Strategies will be tailored to help address barriers and enhance facilitators to PA promotion identified by each team. All teams will receive access to an online education and training resources hub and be offered a choice of additional implementation strategies (table 2).

**Table 2:** Implementation strategies to be tailored to each clinical team for the PROMOTE-PA study

Implementation strategy	Mode of delivery/where/length	Content
Education and training	Online resource hub accessible through website. All clinical teams in the intervention group will be given access to the online resource hub. Approximate total time to review all content=4 hours. Clinical teams will also be offered the option of face-to-face education and training	The online resources contain information, case studies and training videos supporting each of the different physical activity promotion options (coaching, referral, prescription and transition programmes). Additional resources include: Short educational videos/webinars presenting simulated clinical scenarios of health professionals delivering physical activity promotion targeting knowledge gaps identified in PROMOTE-PA part 1 pre-implementation study Links to health professional and patient-facing resources on physical activity benefits, disease-specific considerations, examples of different physical activity options. Resources will include links to available resources, eg, Moving Medicine: https://movingmedicine.ac.uk/; WHO: https://www.who.int/health-topics/physical-activity; and copies of study developed resources
Tailored strategies to address community referral barriers	Referral strategies will be tailored and determined with each clinical team to address their specific context and the barriers they have with community referrals for their patients	Example referral strategies include: An Activity Directory is available in the online resource hub. It provides a comprehensive geographical map and list of community physical activity opportunities across the included local health districts Provide training in finding physical activity opportunities, help to develop links with physical activity providers, develop referral resources Explore and develop new models of service delivery where indicated to support patients transitioning from hospital-based to community-based physical activity opportunities. These programmes could use existing staffing and partnerships with community physical activity facilities Develop systems of referral to the PROMOTE-PA Linkage Programme (developed specifically for this study) and new physical activity programmes that support patients in transitioning from hospital-based to community physical activity programmes. Integrate these new referral systems into the clinical workflows for the team
Experts and clinical mentors (external to clinical team)	Mixed delivery—online and face-to-face options. Tailored to each team	Could include presentations and training (eg, how to do physical activity promotion, advice on specific considerations when promoting physical activity to different populations, how to navigate health professional time constraints) and Q&A sessions/discussions on incorporating physical activity promotion into clinical practice
Clinical champions (internal member of clinical team)	Mix of face-to-face and online support delivered to clinical champion, tailored to each team	Identifying and supporting clinical champions to drive implementation of physical activity promotion into routine practice. Physical activity champions could be identified at the individual team level or site-wide depending on contextual factors. Support offered could include identifying/modifying/developing resources for their team (eg, enhancing connections with local community physical activity options), identifying and connecting with appropriate physical activity opportunities, and modifying clinical assessment forms to include physical activity information to collect

This table has previously been published in the effectiveness protocol for PROMOTE-PA, which can be found here: https://doi.org/10.1136/bmjnph-2024-000901. It has also been included in this new publication for reader ease.

PROMOTE-PA, Promotion of Physical Activity by Health Professionals.

### Effectiveness outcomes

Effectiveness outcomes will be collected at baseline, 3 and 6 months post-randomisation. The primary effectiveness outcome will be patients’ self-reported time (hours/week) spent in moderate-to-vigorous physical activity (MVPA) over the last week. Secondary outcomes will include the self-reported number of days per week participating in muscle-strengthening activity and balance/functional training for adult participants. The number of days per week active for more than 60 min will be assessed for children/adolescents. Physical functioning, global perceived change in PA and mobility, and utility-based quality of life will also be measured.

### Programme theory

The primary aim of the PROMOTE-PA intervention is to improve PA levels in patient participants receiving outpatient or community-based care through supporting clinical teams in promoting PA using implementation strategies.

Previous studies show that health professionals view PA promotion as a part of their role.^9 10^ However, while some PA promotion is currently taking place, it is not routinely incorporated into standard practice.^12^ Given the opportunities health professionals have to deliver tailored advice with authority at critical points in people’s healthcare journeys, efforts to improve the amount and quality of PA promotion in routine practice should be targeted.^19^ We hypothesise that by understanding and addressing barriers to PA promotion identified by health professionals, we will influence key drivers of behaviour change—that is, capability, opportunity and motivation (the COM-B model).^20^ Furthermore, by tailoring implementation strategies to address specific barriers faced by clinical teams, it is expected that health professionals will promote PA to their patients more often and more effectively, leading to an increase in PA participation in individuals receiving outpatient and community-based care.

### Intervention design

The 5As intervention approach that health professionals will be encouraged to use is underpinned by theoretical models of behaviour change: the COM-B model,^20^ Self-determination theory^21^ and Social Cognitive theory.^22^ It includes behaviour change techniques shown to increase PA in the general population^23^ and people with physical disabilities.^24^ A motivational interviewing communication style will also be encouraged to elicit and strengthen patients’ motivation for change.^25^

### Implementation strategies design

The development of the implementation strategies was guided by the findings of previous quantitative and qualitative research^9 11 12 26^ and formative work (PROMOTE Part 1) completed as part of the overall PROMOTE-PA study to understand the current, context-specific experience of health professionals in delivering PA promotion. The completed formative qualitative work will be published separately.

The Consolidated Framework for Implementation Research (CFIR)^27^ was used to map existing and potential barriers and facilitators to implementation within the intervention contexts (table 3), identified through previous surveys and interviews with clinicians across three local health districts in NSW.^9 12^ For example, inner contextual (health service level) barriers included competing clinical demands, limited access to information about suitable PA programmes and a lack of knowledge of PA guidelines.^9 11 12^ Outer contextual barriers included patient costs and lack of suitable community-based PA programmes. Using the CFIR-Expert Recommendations for Implementing Change (ERIC) matching tool, these barriers and facilitators were matched to potential implementation strategies from the ERIC taxonomy.^28^ As part of PROMOTE Part 1, health professionals identified local barriers and facilitators to PA promotion and both co-designed and adapted implementation strategies for their different clinical contexts to be used in the trial (table 2). The emergence of new implementation strategies and adaptations made to the existing strategies will be reported as part of the process evaluation.

**Table 3:** Constructs identified by clinical teams as barriers and facilitators to physical activity promotion using the Consolidated Framework for Implementation Research (CFIR) 1.0

Domains/constructs	Relevance in PROMOTE-PA
Intervention characteristics	
Intervention source	Choice by teams (f)
Evidence strength and quality	Lack of knowledge (b)
Relative advantage	Competing demands (b) Benefits of increased physical activity for patients (f)
Adaptability	Tailoring of approach and choice of implementation strategies to suit local needs (f)
Complexity	Time, resource and knowledge requirements (b)
Cost	Opportunity cost (b)
Implementation planning and processes	
Planning	Work completed in Part 1 of PROMOTE-PA (f)
Engaging	Engaging with clinical teams/health professionals in Part 1 of PROMOTE-PA (f)
Opinion leaders	Engagement with key leaders at sites (f)
Champions	Plan to identify champions within clinical teams (f)
External change agents	PROMOTE-PA team providing implementation support to clinical teams (f)
Characteristics of individuals: health professionals	
Knowledge and beliefs about the intervention	Existing knowledge gaps (b) Primarily positive beliefs about physical activity promotion (f)
Self-efficacy	Overestimation of skills and knowledge (b)
Individual stage of change	Varied individual stages of change (b)
Individual identification with organisation	Recent lack of individual identification within organisation (b)
Other personal attributes	Commitment to high quality, patient-centred care (f)
Inner setting: clinical settings in NSW	
Structural characteristics	Large, siloed, staff changes (b)
Networks and communication	Identified as potential (b) (f)
Culture	Commitment to high quality care (f)
Implementation climate	Impacts of COVID-19 pandemic (b)
Tension for change	Physical activity promotion practices not being a priority for change (b)
Compatibility	Tailoring and interest among teams (f)
Relative priority	Many existing priorities (b)
Organisational incentives and rewards	Difficulty in provision of organisational incentives and rewards (b)
Learning climate	Work within workflows identified as both a (b) (f)
Readiness for implementation	Organisational commitment to partnership grant (f)
Leadership engagement	Engagement in project (f)
Available resources	Partnership agreement and fit into workflow (f)
Access to knowledge and information	Research team partnership grant (f)
Outer setting: community physical activity settings	
Patient needs and resources	Recognition of the need for appropriate cost, transport, suitable options, patient motivation and online options (f)
Cosmopolitanism	Poor links between public health system and community (b)
External policies and incentives	Poor regulation of accessibility of community settings, services starting and stopping, variable cost of services (b) Availability of some lower cost/subsidised options (f)

(f): facilitator, (b): barrier.

NSW, New South Wales; PROMOTE-PA, Promotion of Physical Activity by Health Professionals.

### Logic model

**Figure 1:**
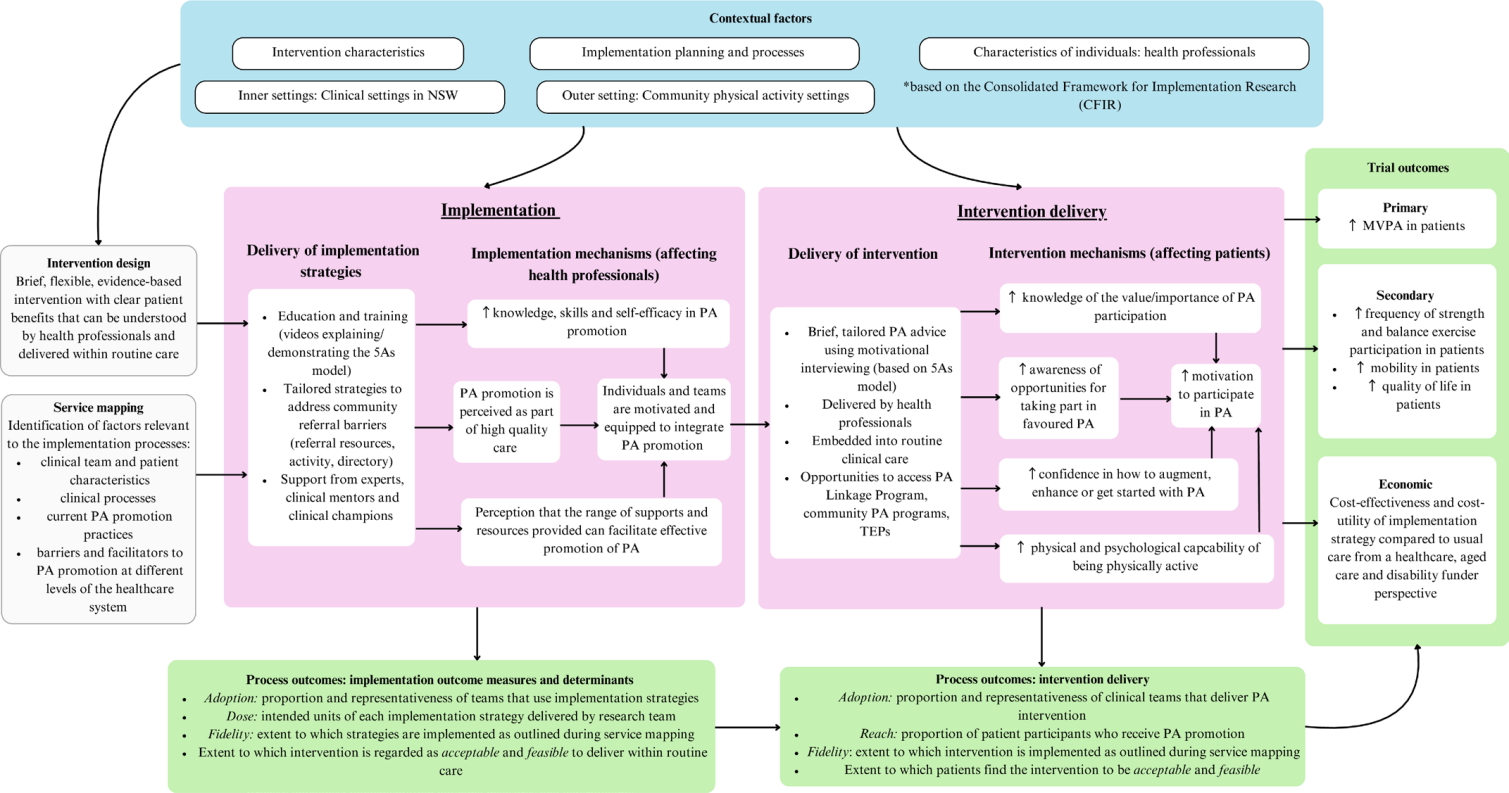
Promotion of Physical Activity by Health Professionals implementation trial logic model. CFIR, Consolidated Framework for Implementation Research; MVPA, moderate-vigorous physical activity; NSW, New South Wales; PA, physical activity; TEP, transition exercise programme; 5As, Assess, Advise, Agree, Assist, Arrange.

A logic model depicting the implementation strategies and intervention informed by their underlying programme theory, and the expected impact of effectiveness and implementation outcomes is presented in figure 1. This model is informed by the United Kingdom’s Medical Research Council framework for linking implementation, mechanisms of impact and context within a process evaluation.^29^ Our process evaluation will test this logic model by capturing information in relation to clinical context, implementation determinants and outcomes, to understand the extent to which our implementation strategies worked, and how and why they may have worked.

## Methods

A mixed-methods process evaluation will be conducted alongside the PROMOTE-PA trial,^15^ informed by McKay’s evaluation roadmap of implementation outcome measures and determinants,^30^ and hybrid effectiveness implementation study design principles.^31^

### Data collection

Data on implementation outcome measures and determinants^30^ (online supplemental additional file 1) will be collected at two levels: the delivery of PA promotion by health professionals to their patients and the delivery of implementation strategies by the research team to health professionals.

### Quantitative data collection

#### Intervention level

For patient participants in the intervention group, a self-reported study-specific *Impressions of Healthcare* survey will be used to determine the proportion of patients who received PA promotion, and their experience with receiving PA promotion from health professionals, within the past 6 months.

#### Implementation strategies level

For health professional participants in both groups, data will be collected on current PA promotion behaviours, clinical experience (years) and PA participation, to characterise the clinical teams in relation to implementation measure outcomes (online supplemental additional file 1). Health professionals will also be asked to estimate how often they deliver elements of PA promotion within their usual practice, on a 5-point Likert scale from ‘never’ to ‘always’. Data will be collected using a baseline survey, with the PA promotion questions also asked in a follow-up survey distributed at the end of the implementation support period (intervention group) or after patient recruitment has concluded (control group).

For health professionals in the intervention group, data on delivery of PA promotion will be captured using a self-reported clinician checklist of PA promotion elements using the 5As model at baseline and 6 months. Each health professional will be asked to complete this for approximately five consecutive patients, indicating whether the elements of the 5As model were delivered during each consult. The impact of implementation support on health professionals will be assessed using a *Physical Activity Promotion Support Survey* following the implementation support period.

Study records will capture data relating to adoption, dose and reach of, and fidelity to implementation strategies delivered to clinical teams at the end of the implementation support period or when a health professional departs a team. A medical record audit will be conducted to assess the delivery of PA promotion to patients at baseline compared with 6 months.

### Qualitative data collection

#### Intervention level

Semi-structured interviews will be conducted with 20–30 patient participants (aged ≥13 years), and parents and carers. Purposive sampling will target a mix of patients from different geographical areas, client populations (age, gender, socioeconomic background, language spoken at home) and clinical settings. Interviews will be conducted at the 6-month follow-up. Interviews will take 30–40 min.

#### Implementation strategies level

Interviews will be conducted at 5–6 sites with 20–30 health professionals (including key liaison personnel at the sites) to evaluate their experience with and perceptions of receiving implementation support from the research team. Purposive sampling will be used to maximise heterogeneity of health professionals from different local health districts, private practices and clinical settings, who used a variety of implementation strategies and options for PA promotion. Interviews will be conducted at 6 months following provision of implementation support. Interviews will take up to 40 min.

Interviews may be conducted face-to-face or online, and focus groups may be offered to health professionals as an alternative. All interviews and focus groups will be audio-recorded. Audio-recordings will be professionally transcribed and checked for accuracy by a research team member present for data collection. Field notes may be taken by the researcher throughout or immediately after the interviews, to be later incorporated into the transcripts.

### Record of tailoring and adaptations

The FRAME-IS framework will be used to document the tailoring of implementation strategies to clinical teams (eg, providing an option of an online channel to share resources to accommodate the clinical team’s workflow)^32^ throughout the period of implementation support. This will include when the adaptation occurred and a description of the rationale for these changes. Data will be drawn from implementation logs kept by the research team. Implementation logs will detail the planning, delivery and follow-up of implementation support provided (including time taken, elements of implementation plan discussed at meetings with clinical teams).

### Scalability assessment

A preliminary assessment of scalability will be conducted and reported using the Intervention Scalability Assessment Tool (ISAT) (online supplemental additional file 2).^14^ It is expected that our initial assessment of scalability may evolve based on adaptations to implementation support elements and intervention delivery throughout the trial, and the trial outcomes. An updated version of the ISAT tool will be presented on completion of the trial.

### Data analysis

#### Quantitative analysis

Survey data will be managed using the University of Sydney Research Electronic Data Capture. Data for the recruitment, and training and resources log, will be recorded in Microsoft Excel spreadsheets by the research team.

Data on the implementation measures and determinants will be summarised using descriptive statistics. Google Analytics will be used to generate descriptive analytics on the usage of the online training and resources hub and the activity directory.

#### Mediation analysis

In evaluating the implementation strategies, interviews and focus groups will be conducted to improve our understanding of how and why the strategies did or did not work. These findings will be considered alongside quantitative data collected, to inform the implementation measures and/or determinants to be evaluated using a causal mediation analysis. The exact causal model will be selected based on the qualitative feedback and will be pre-defined before the mediation analysis is performed. Mediation analysis will seek to identify the causal mechanisms through which the suite of implementation strategies impacts PA levels.

#### Qualitative analysis

A qualitative descriptive approach^33^ will be taken to managing and analysing the data from participant interviews. This will allow learnings from the interviews to be applied to target improvements in healthcare service provision through the informed adaptation of the implementation strategies and intervention.^34^ Transcripts will be imported into the qualitative software program NVivo to assist with analysis. Deductive qualitative content analysis^35^ will be used to code the data framed by key constructs in McKay’s evaluation roadmap,^30^ and in the programme theory. Data will also be reviewed inductively and coded to capture any additional themes in the data that are relevant to our research questions. Analysis of the transcripts and detailed field notes will be conducted iteratively throughout the period of data collection. The research team will develop an initial coding framework and two researchers will code some early transcripts independently. Codes will be discussed by the researchers, who will then refine the coding framework as required and apply it to further transcripts, with further revisions to capture variations in the data, if required. When approximately half of the transcripts have been analysed and early themes have been developed, a workshop will be held with other research team members (with multidisciplinary backgrounds) who will serve as ‘critical friends’ to review the analysis that has been performed, with the goal of refining findings in relation to the road map and programme theory constructs, and any themes generated up to that point. This critical review process will be continued through the process of writing up.

Later stage qualitative analysis will incorporate emergent findings from the quantitative data analysis to develop a nuanced picture combining narrative accounts with descriptive statistics that offer insights into how and why implementation support and the use of the 5As intervention influenced key drivers of behaviour change, or why they did not. This will help us understand variations in outcomes and identify context-sensitive practice recommendations for supporting and promoting PA in healthcare settings.^36^

## Discussion

This protocol describes the plan for a mixed-methods process evaluation to be conducted alongside the PROMOTE-PA trial, framed using McKay’s evaluation roadmap.^30^ Given the complexity of the programme, this approach will allow us to understand the mechanisms and factors driving the influence of the selected implementation strategies. This is especially important considering the diverse range of settings and clinical teams that may be engaged in future implementation efforts. In line with guidance for process evaluations of complex interventions,^29^ publication of this protocol ensures transparency in our methodology and the consequent interpretation of the results of this evaluation. However, we do recognise the importance of inbuilt flexibility as part of this process evaluation to ensure all relevant and important implementation factors are captured and evaluated.

We recognise some limitations to our process evaluation. First, while we are collecting the data on the delivery of the 5As in both groups, implementation data for most implementation determinants will only be collected for health professionals and patients assigned to the early implementation support group. We have limited the collection to the early implementation support group for pragmatic reasons, including optimising the use of available trial resources and managing participant burden. We expect that the level and quality of implementation support provided by the research team will be the same between the groups. Furthermore, we will undertake representative sampling to ensure a variety of perspectives among the important stakeholder groups are captured through qualitative interviews with the early implementation support group.^37^

Second, we are relying mainly on self-reported data from health professionals relating to their PA promotion activity, subjecting the data to the risk of reporting bias. While direct observation of health professionals may be more accurate, this would mean additional health professional burden and resources required from the research team. To supplement self-reported data, we intend to conduct audits of medical records (ie, health professionals’ notes).

Despite these limitations, a comprehensive approach is being taken in evaluating a range of implementation measures and determinants using a mixed-methods approach. Evaluation of these process outcomes at both the level of the implementation strategies and intervention delivery will allow for an in-depth understanding of the implementation strategies from a qualitative perspective, supported by quantitative data. Data from the process evaluation will support the interpretation of effectiveness outcomes and provide insight into the mechanisms through which implementation strategies to support PA promotion by health professionals influence PA participation among patients. This insight allows for further refinement of programme theory, to inform and support future implementation efforts.^38 39^

While the literature informing the benefits of PA is extensive, there remains a gap in the translation of these findings into clinical practice and policy. Implementation research such as the present trial will contribute to addressing this gap, informing effective strategies to support PA promotion as part of routine practice. Process evaluation is a valuable tool for evaluating implementation outcomes and understanding contextual factors that affect local implementation, which have been identified by practitioners and policy makers as important components in informing scale-up.^40^ Findings of this process evaluation will inform broader implementation and scale-up of PA promotion by health professionals to improve PA participation among patients seeking healthcare.

### Trial status

The trial is currently underway, with recruitment ongoing (having commenced in January 2024).

## Supplementary material

10.1136/bmjnph-2025-001193 online supplemental file 1

10.1136/bmjnph-2025-001193 online supplemental file 2

## Data Availability

Data sharing not applicable as no datasets generated and/or analysed for this study.
