# Supplementary material for: Processes of supported promotion of physical activity by health professionals: protocol for mixed-methods evaluation within the PROMOTE-PA hybrid effectiveness-implementation cluster randomised controlled trial
Source: BMJ Nutr Prev Health. 2025 Aug 24;8(2):e001193. doi: 10.1136/bmjnph-2025-001193 (PMC12936534; doi:10.1136/bmjnph-2025-001193)
Supplement: online supplemental file 1 [file bmjnph-8-2-s001.pdf]

Additional File 1. Implementation measures and determinants to be collected in the PROMOTE-PA study (intervention group only) based on McKay's evaluation roadmap.

Implementation Measures	Delivery of the intervention			Delivery of the implementation strategies		
	**Definition**	**Measurement tool**	**Timepoint**	**Definition**	**Measurement tool**	**Timepoint**
1. Adoption	Proportion and representativeness of clinical teams that deliver PA intervention.	Characteristics and proportion of health professionals delivering the intervention within included clinical teams assessed by study-specific baseline and follow-up survey, self-reported by health professionals.	Baseline, 6 months post-implementation commencement	Proportion and representativeness of the sites that utilise implementation strategies.	Proportion of staff in clinical teams that utilise the implementation strategies, evaluated using implementation logs kept by research team	6-months post-implementation commencement.
2. Dose	Intended units of each intervention component delivered to participants by the clinical team.	*Not assessed as PA promotion is tailored to each site.*	*N/A*	Intended units of each implementation strategy delivered by the research team.	Average dose (hours) per clinical team of implementation strategies delivered, measured using a study-specific training and resources log.	6-months post-implementation commencement.
3. Reach	Proportion of patient participants who receive PA promotion.	1. Medical record audit assessing delivery of PA promotion to patients.<br>2. Proportion of patients who received PA promotion, measured by patient self-reported study specific survey assessing their impressions of healthcare over the previous 6 months.	1. Baseline, 6 months post-implementation commencement<br>2. 6 months patient follow-up	Proportion of clinical teams that participate in the implementation support.	Proportion of the clinical teams who participated in the study and received the implementation strategies compared to the number who were identified by the research team and invited to participate, assessed by an audit of study records maintained by research team.	Following recruitment of final clinical team.
4. Fidelity (adherence)	The extent to which an intervention is implemented as was outlined and agreed in	Proportion of health professionals who deliver PA promotion, measured by self-reported clinician checklist of	Baseline, 6 months post-implementation commencement	The extent to which implementation strategies are implemented as agreed	Proportion of implementation strategies used by each clinical team compared with the strategies identified in the implementation	At conclusion of implementation support period.

	the service mapping document at the start of the intervention period – and delivered by the clinical team.	PA promotion strategies using the 5As model, as per each team's implementation plan developed during the implementation period.		and outlined in the service mapping document – and delivered by the research team.	plan. Documented by research team on a checklist tailored for each clinical site.	
**Implementation Determinants**	**Delivery of the intervention**			**Delivery of the implementation strategies**		
	**Definition**	**Measurement tool**	**Timepoint**	**Definition**	**Measurement tool**	**Timepoint**
1. Context	Aspects of the larger social, political, and economic environment that may influence intervention implementation.	Research team and clinical teams to map relevant contextual issues using the service mapping form.	Service mapping period.	Aspects of the larger social, political, and economic environment that may influence delivery of the implementation strategies	Research team and clinical teams to map relevant contextual issues using the service mapping form.	Service mapping period
2. Culture	Organisations' norms, values, and basic assumptions around selected health outcomes.	Clinicians' perceptions on the importance of delivering PA promotion, assessed by:<br>1. Study-specific survey<br>2. Semi-structured face-to-face interviews with health professionals.	1. At conclusion of implementation support period<br>2. 6-months post-implementation commencement.	Organisations' norms, values, and basic assumptions around selected implementation strategies.	Semi-structured interviews with health professionals and managers.	6-months post-implementation commencement.
3. Acceptability	Perceptions among the clinical teams that a given intervention is agreeable, palatable, or satisfactory.	Qualitative semi-structured face-to-face interviews with health professionals.	6-months post-implementation commencement.	Perceptions among the clinical teams that implementation strategies are agreeable, palatable, or satisfactory.	Acceptability of PA promotion support strategies assessed by study-specific survey of health professionals.	At conclusion of implementation support period.
4. Adaptability	Extent to which an intervention can be adapted, tailored, refined, or reinvented to meet local needs.	1. Number of different PA promotion interventions offered to clinical teams, assessed by site-specific study records.	6-months post-implementation commencement.	Extent to which implementation strategies can be adapted, tailored, refined, or reinvented to	Research study team to evaluate number of different ways support to deliver PA promotion was offered to clinical teams, using site-specific implementation logs kept by the	At conclusion of implementation support period.

		2. Qualitative semi-structured face-to-face interviews with health professionals.		meet the needs of organisations at scale-up.	research team to be mapped to the FRAME-IS framework.	
5. Feasibility	Perceptions among the clinical teams that an intervention can be successfully used or carried out within a given organisation or setting.	Qualitative semi-structured face-to-face interviews with health professionals.	6-months post-implementation commencement.	Perceptions among the clinical teams that implementation strategies can be successfully used or carried out at scale within different organisations or settings.	Qualitative semi-structured face-to-face interviews with health professionals.	6-months post-implementation commencement.
6. Compatibility	Extent to which an intervention fits with the mission, priorities, and values of organisations or settings.	Semi-structured face-to-face interviews of health professionals assessing how the delivery of PA promotion fitted in with clinical workloads.	6-months post-implementation commencement.	Extent to which implementation strategies fit with the mission, priorities, and values of organisations at scale-up.	Semi-structured face-to-face interviews of health professionals assessing how the delivery of implementation support fitted in with clinical workloads.	6-months post-implementation commencement.
7. Cost	Money spent on design, adaptation and implementation of an intervention.	Trial based site-specific records for delivery of the PA promotion interventions.	Duration of the study period up to 6-month follow-up of patient participants.	Money spent on design, adaptation and delivery of implementation strategies.	Trial based site-specific records for delivery of the implementation strategies.	Duration of the study period up to 6-month follow-up of patient participants.
8. Satisfaction	Clinical teams' satisfaction with an intervention and with interactions with the support system.	Semi-structured face-to-face interviews of health professionals assessing satisfaction with PA promotion interventions.	6-months post-implementation commencement.	Clinician teams' satisfaction with implementation strategies.	Semi-structured face-to-face interviews of health professionals assessing satisfaction with implementation strategies.	6-months post-implementation commencement.
9. Complexity	Perceptions among the clinical teams that a given intervention is relatively difficult to understand and use; number of different	Semi-structured face-to-face interviews with health professionals assessing their perception of complexity of the PA promotion interventions.	6 months post-implementation commencement	Perceptions among the clinical teams that implementation strategies are relatively difficult to understand and use; number of	Semi-structured face-to-face interviews with health professionals assessing their perception of complexity of the implementation strategies.	6 months post-implementation commencement

	intervention components.			different strategies. Related to implementation setting.		
10. Self-efficacy	Clinical teams' belief in their ability to execute courses of action to achieve implementation goals.	Study-specific survey of health professionals assessing self-confidence in ability to deliver PA promotion.	6 months post-implementation commencement	Clinical teams' belief in their ability to execute courses of action to achieve implementation goals.	Semi-structured face-to-face interviews with clinical team leaders assessing belief in ability to achieve implementation goals.	6 months post-implementation commencement

PA: physical activity
