# Supplementary material for: Processes of supported promotion of physical activity by health professionals: protocol for mixed-methods evaluation within the PROMOTE-PA hybrid effectiveness-implementation cluster randomised controlled trial
Source: BMJ Nutr Prev Health. 2025 Aug 24;8(2):e001193. doi: 10.1136/bmjnph-2025-001193 (PMC12936534; doi:10.1136/bmjnph-2025-001193)
Supplement: online supplemental file 2 [file bmjnph-8-2-s002.pdf]

The Australian Prevention Partnership Centre
Systems and solutions for better health

Intervention Scalability Assessment Tool

January 2019

Intervention Scalability Assessment Tool

Prepared by: The Australian Prevention Partnership Centre and NSW Ministry of Health

Contributing authors:

The Australian Partnership Prevention Centre, NSW Ministry of Health, University of Sydney, University of Newcastle

Dr Andrew Milat
Ms Karen Lee
Dr Anne Grunseit
Dr Kathleen Conte
Associate Professor Luke Wolfenden
Professor Adrian Bauman

© Sax Institute 2017
This work is copyright. It may be reproduced in whole or in part for study training purposes subject to the inclusions of an acknowledgement of the source. It may not be reproduced for commercial usage or sale. Reproduction for purposes other than those indicated above requires written permission from the copyright owners.

Enquiries regarding this report may be directed to:

The Australian Prevention Partnership Centre
www.preventioncentre.org.au
Email: preventioncentre@saxinstitute.org.au
Phone: +61 2 9188 9520

Our funding partners

Australian Government — National Health and Medical Research Council
Australian Government — Department of Health
ACT Government — ACT Health
Cancer Council Australia
NSW Government
Government of South Australia — SA Health
Tasmanian Government
VicHealth

Hosted by

saxinstitute
Supporting smart decisions. Powered by research.

Contents

Intervention Scalability Assessment Tool 2

1 Introduction 4

1.1 What is scale up and scalability, and why is it important? 4
1.2 Purpose and objectives of the Intervention Scalability Assessment Tool (ISAT) 4
1.3 Limitations 5
1.4 How was the ISAT developed? 5

2 How to complete the ISAT 6

2.1 Information sources 6
2.2 Process for completing the ISAT 6
2.3 Structure of the ISAT 6
2.4 How to complete the questions 7
2.5 Scoring the readiness questions at the end of each domain 7

3 PART A: SETTING THE SCENE 9

3.1 Proposed level and method of scale up 9
3.2 DOMAIN A1: THE PROBLEM 10
3.3 DOMAIN A2: THE INTERVENTION 11
3.4 DOMAIN A3: STRATEGIC/POLITICAL CONTEXT 12
3.5 DOMAIN A4: EVIDENCE OF EFFECTIVENESS 13
3.6 DOMAIN A5: INTERVENTION COSTS AND BENEFITS 15

4 PART B: INTERVENTION IMPLEMENTATION PLANNING 16

4.1 DOMAIN B1: FIDELITY AND ADAPTATION 16
4.2 DOMAIN B2: REACH AND ACCEPTABILITY 18
4.3 DOMAIN B3: DELIVERY SETTING AND WORKFORCE 20
4.4 DOMAIN B4: IMPLEMENTATION INFRASTRUCTURE 22
4.5 DOMAIN B5: SUSTAINABILITY 23

5 PART C: SUMMARY OF SCALABILITY ASSESSMENT 25

ATTACHMENT A: REPORT TEMPLATE 27

References 28

1 Introduction

1.1 What is scale up and scalability, and why is it important?

Scaling up is defined by the World Health Organization as *'the deliberate effort to increase the impact of successfully tested health interventions so as to benefit more people and to foster policy and program development on a lasting basis'*. (1) 'Scale up' ranges along a continuum, from increasing the reach and adoption of a local program to a greater number of sites, through to regional, state-wide, national or even the international roll-out of an intervention. Scaling up evidence-based population health interventions is necessary to ensure maximum benefit from effective health services and programs at the population level.

Scalability, on the other hand, is defined as *"the ability of a health intervention shown to be efficacious on a small scale and/or under controlled conditions to be expanded under real world conditions to reach a greater proportion of the eligible population while retaining effectiveness". (2)* The scalability of a program or intervention[1] will vary depending on the context and the design of the program itself. Various factors require consideration when assessing the scalability of any program or intervention and deciding whether or not it should be implemented at scale.

1.2 Purpose and objectives of the Intervention Scalability Assessment Tool (ISAT)

The ISAT (the 'tool') is designed to assist practitioners, policy makers, program managers and researchers to determine the scalability of a discrete health program or intervention. Though developed for use in the population health context, the ISAT is adaptable and could be used in other human service settings.

The ISAT has primarily been designed to:

- Assist in making assessments of the scalability of an intervention
- Identify and assess contextual factors helping or hindering scale up
- Provide a mechanism for identifying any gaps in the information required to make an assessment or to inform an assessment on scalability of an intervention
- Provide a structure for thinking through some of the key considerations for determining if a program or intervention is scalable.

The tool however, may also be used for:

- The pre-design process, when considering how to design a program and/or intervention for implementation and/or future scale up
- Making comparisons across multiple programs and/or interventions being considered for scale up
- Making assessments on the readiness of an intervention for scale up
- Structuring the thought processes for making decisions on whether to fund or scale up new/pilot interventions
- Deciding on the future of current interventions, i.e. whether to continue or to terminate an intervention
- Informing the strategic planning processes.

By using this tool, the information collected and assessments made about the program and/or intervention can be documented and stored for future use. This may also provide a basis to inform future decisions and planning processes and/or enable a repository of information which can be used for retrospective reflection.

---

[1] Throughout this document, the terms 'program' and 'intervention' will be used interchangeably.

1.3 Limitations

This tool lends itself to application in most health- and human services-related programs/interventions. However, not every population health intervention, program or even policy requires a scalability assessment. There may be legitimate reasons why policy action may occur in the absence of direct causal evidence of program/intervention effectiveness. For example, it may not be possible to generate definitive causal evidence of the effect of legislative change prior to implementation, however, if the possible impact of change is great and the cost are low, there may be merit in implementing such changes.

1.4 How was the ISAT developed?

The tool was developed through a review of the implementation science literature and several rounds of input from implementation researchers, policy makers and practitioners actively involved in program management and/or the scaling up population health interventions and programs.

2 How to complete the ISAT

2.1 Information sources

A variety of information sources should be used to complete the ISAT and may include:

- Available evaluation reports
- Published literature (for example, academic journals)
- Grey literature (for example, published or unpublished reports, white papers and unpublished evaluations)
- Practice based information (for example, local strategic plans, practice manuals, protocols, clinical guidelines)
- Expert opinion.

Where information is missing or unavailable, a judgement is required regarding:

- The importance of the knowledge gap to decision making
- Whether the gaps can be addressed during implementation
- Whether further research is required before an intervention is to be considered for scale up.

All information sources should be referenced and documented in response to the questions posed in the tool for future reference.

2.2 Process for completing the ISAT

The questions posed in the ISAT are numerous and wide-ranging. However, the intent is to stimulate thinking and promote active consideration of factors that have been shown to be important when assessing scalability. It may not be possible to answer all the questions, but the absence of information can be an important finding in itself.

We recommend that the ISAT be completed by a group of stakeholders collectively. As a variety of information sources should be used as part of the process, one individual is unlikely to have access to all the information required. An ideal team of stakeholders will reflect a range of expertise in research, program planning, implementation and practice. For example, research expertise will facilitate assessments as to the strength of the evidence, while practitioner/policy maker experience will be critical in identifying important contextual factors that could affect implementation and the scale up process.

A summary reporting template has been developed to assist in presenting the key findings of the assessment. This includes a recommendation regarding scale up for consideration by senior decision makers.

If a decision is made to scale up an intervention, the information gathered by this tool can be used to develop a comprehensive scale up plan. The NSW Health Guide to Scaling Up Population Health Interventions (3) describes a comprehensive step-by-step planning process for the scale up of interventions.

2.3 Structure of the ISAT

The tool consists of three parts. Within each the first two parts are five 'Domains'. The purpose of PART A is to provide background information on the public health problem, the context within which it is proposed that the intervention will be scaled up, and a description of the intervention.

The purpose of PART B is to consider implementation and feasibility factors relating to all aspects, including fidelity and adaptations, reach and acceptability, delivery settings and agents, as well as implementation infrastructure and training.

PART C provides an opportunity to summarise all the information gathered to facilitate the process of making a recommendation on scalability.

2.4 How to complete the questions

Determining future scalability of any intervention involves the active consideration of:

a) Current context
b) How the intervention is currently implemented, and
c) How implementation may need to be modified for the intervention to work at scale.

Some of the questions may require you to make assumptions and forward projections, which may or may not be possible depending on your context and information at your disposal. It is not compulsory to make these projections; however, the aim is to prompt thinking about any gap between the current approach to implementation and implementing at scale.

NOTE: Although *some* aspects of implementation scale up planning have been addressed in this tool, it is worth noting that the ISAT tool is focused on assessing the *scalability of the intervention* at this stage. Once a decision has been made that the intervention merits scaling up, the next step will be to develop a detailed implementation scale up strategy (3). The considerations made, and information gathered for completing this tool, will be useful for the development of the future implementation scale up strategy should this be warranted.

2.5 Scoring the readiness questions at the end of each domain

At the end of each domain are several optional questions that provide a readiness assessment of the information provided for that domain. The purpose of these readiness questions is to assist in identifying the strengths and weaknesses across the domains. Each question can be scored from 0–3, where the minimum score for each domain is 0 and the maximum score is 3. In order to derive a final score for the domain, take the average score across the questions (if there is more than one question). If there are multiple people providing a score, the total score by domain can be summed and averaged across the number of scorers. If the question is not applicable, it does not need to be scored and the response 'N/A' can be selected. *It is important to note that there is no 'total' or combination score that can be derived from scoring all the individual domains*. The omission of such a total score is deliberate, as it is recognised that the not all the domains are necessarily equal across all contexts and scenarios. Low scores in some domains may be acceptable in some situations, but not so in others, depending on the perceived importance of the domain in the context of intervention for scale up. Once the score per domain is derived, it can be entered into the accompanying Excel spreadsheet to plot the scores on the graph and provide a visual representation across all domains to enable a comparison. This graph will highlight the domains which may need to be strengthened or improved. It also provides a starting point to facilitate discussions on the potential scalability and/or readiness of the intervention in question. It may also promote the development of strategies and/or solutions to mitigate any potential issues that may arise during the scaling up phase.

Figure 1 below provides an illustration of the graph

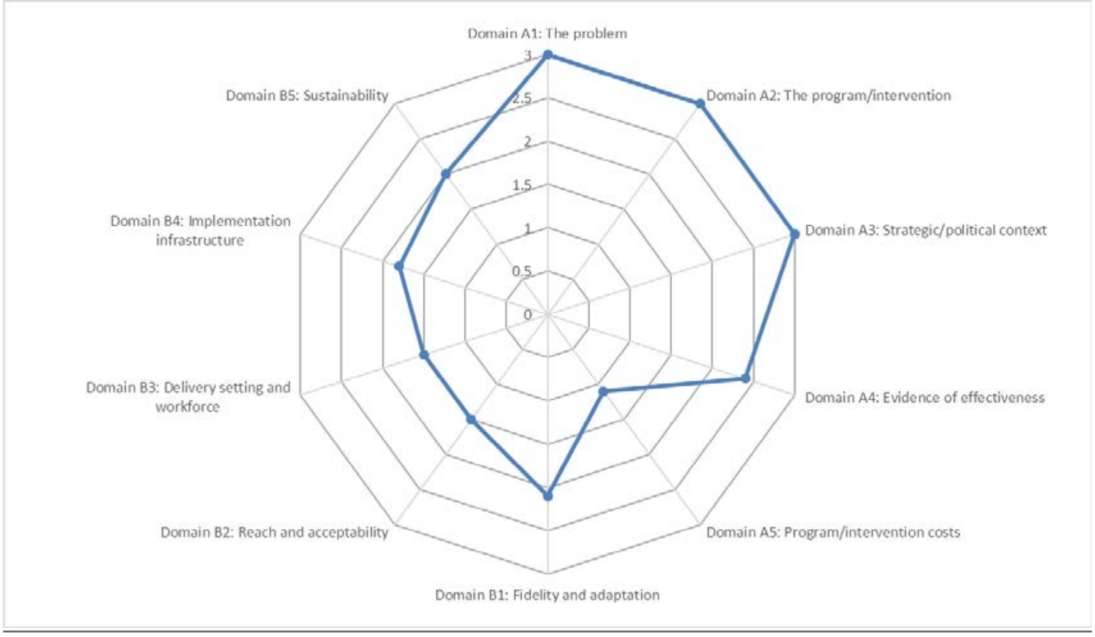

Figure 1 above provides an illustration of the graph once the scores have been entered by domain. From this example it can be noted that several domains scored quite high while other domains scored considerably lower. From this example, it is immediately apparent, where some of the potential strengths and weaknesses are of the program/intervention following the scalability assessment. This result can be used to promote further discussion or action as to the potential scalability of the program/intervention.

3 PART A: SETTING THE SCENE

3.1 Proposed level and method of scale up

Name of Intervention being considered: Promotion of Physical Activity by Health [illegible]

The following two overarching questions set the scene for the completion of the tool. They relate to the level of scale up and the likely scale up pathway being considered.

Question 1: What is the <u>ultimate</u> level of scale up you are trying to achieve? (Select as many as necessary)

☐ Scaled up across multiple sites within a region
▣ Scaled up across a local region or province
▣ State or large jurisdiction wide scale up
☐ National or country level scale up
☐ Other level of scale up *(please describe)*________________________________
☐ Don't Know

Question 2: How are you proposing to scale up?

☐ Vertical approach (Simultaneous approach)

*NB: Scaling up using a **vertical approach** involves the introduction of an intervention simultaneously across a whole system and results in institutionalisation of a change through policy, regulation, financing or health systems change (3)*

▣ Horizontal scale up (Stepwise approach)

*NB: Scaling up using a **horizontal approach** involves the introduction of an intervention across different sites or groups in a phased manner, often beginning with a pilot program, followed by stepwise expansion, learning lessons along the way to help refine further expansion (3).*

☐ Other ____

3.2 DOMAIN A1: THE PROBLEM

This domain considers the problem that is being addressed. In this domain, describe the problem, who it affects, what it affects and how it is currently being addressed (if at all). Where possible, draw on any recent data available that provides evidence of the problem and its impacts. This may include population survey data either at the local, regional or national level, or secondary data sources as examples.

Domain A1: The problem	
The problem – Describe what is known about the current nature of the problem	
A1.1: What is the problem and who does it affect?<br>Describe the nature and scope of the problem using epidemiological data (e.g. Who is affected? How widespread is the problem? What is known about the causes?)	Low physical activity is a major public health issue globally leading to 5.3 million avoidable<br><br>deaths each year. Almost one in two Australians (43%)
A1.2: How does the problem impact on the health of the population?<br>Describe the impact of the problem on health and wellbeing of the population (e.g. burden of disease and costs to the health system and society)	Physical inactivity is associated with greater risk of poorer health outcomes and chronic conditions. Physical activity is known to be beneficial to the prevention and management of many chronic conditions such as cardiovascular disease, diabetes
A1.3: What is current practice to address the issue?<br>Describe how this problem is currently addressed in the system	Physical activity promotion is a component of healthcare that aims to change physical activity

3.2.1 Readiness assessment Domain A1: The problem

Question	Not applicable	Not at all	To a small extent	Somewhat	To a large extent
1. Is the problem of sufficient concern to warrant scale up of the intervention/program to address it?	N/A	0	1	2	3
Total score for Domain A1			3		

3.3 DOMAIN A2: THE INTERVENTION

This domain requires a description of the proposed program/intervention to address the problem described above. The aims, objectives, key elements and methods of delivery should be documented.

Domain A2: The program/ intervention	
Current situation – Describe what is currently known about the intervention proposed for scale up	
A2.1: Describe the aims/objectives and intended outcomes of the intervention proposed for scale up<br>Provide (1) a summary description of the intervention and (2) its aims and objectives	The Promotion of Physical Activity by Health Professionals (PROMOTE-PA) involves the delivery of a multicomponent implementation strategy supporting teams of health professionals in hospital and community settings to
A2.2: Describe the key elements of the intervention proposed for scale up (including the process of delivery)<br>What are the key intervention components? (e.g. Frequency and intensity of the intervention, etc)	The intervention delivered by health professionals would be physical activity promotion within routine healthcare interactions. Physical activity promotion delivery would be based on the 5As model (Assess, Advise, Agree, Assist,

3.3.1 Readiness assessment Domain A2: Intervention characteristics

Question	Not applicable	Not at all	To a small extent	Somewhat	To a large extent
**2.** Will the outcomes delivered by this intervention address the needs of the target group (and/or) problem?	N/A	0	1	2	3
**Total score for Domain A2**			2		

3.4 DOMAIN A3: STRATEGIC/POLITICAL CONTEXT

This domain requires consideration of the current strategic/political/environmental context. It may also be worthwhile to consider other influences that may contribute to the context such as industry/commercial players or the non-government sector.

Domain A3: Strategic/political context	
Strategic/political context - Describe what is known about the current strategic/political/environment context	
A3.1: Is addressing the problem consistent with national, state or regional policy directions or priorities?<br>(Yes, No, Don't know). Provide evidence where possible to justify assessment)	Yes. As outlined in Australia's Long Term National Health Plan, Preventive Health forms one of the key pillars of this plan, of which one of the priorities is "Build a more active Australia". This goal will be supported by the implementation
A3.2: Is addressing the problem an identified need of funding agencies?<br>Note: Funding in agencies in this case may include central agencies such as NHMRC, Treasury, Cabinet Office, individual departments, non-government organisations or other advocacy groups<br>(Yes, No, Don't know). Consider if there are any targets/indicators/goals at the local or international level that need to be met	Yes, this project has been funded by the National Health and Medical Research Council (Partnership Projects 2021).
A3.3: How well will the intervention proposed for scale up align with the broader strategic and/or political context?<br>Consider if there are any political, strategic or environmental priorities or strategies this intervention might align with. Also consider the influence of industry and private and non-government sector	The Australian government has set a target of reducing physical inactivity by 15% by 2030. This aligns with the current federal government funding update of the Australia physical activity guidelines, which will be released in 2025. The promotion of physical activity by health professionals to their patients presents a unique opportunity for encouraging

3.4.1 Readiness assessment Domain A3: Strategic/political context

Question	Not applicable	Not at all	To a small extent	Somewhat	To a large extent
3. Is addressing the problem consistent with policy/strategic directions or priorities?	N/A	0	1	2	3
4. Will scaling up the intervention be strategically useful to funders/funding agency?	N/A	0	1	2	3
**Total score for Domain A3**			6		

3.5 DOMAIN A4: EVIDENCE OF EFFECTIVENESS

This domain considers the level of evidence available to support the scale up of the proposed intervention. This includes the consideration of evidence from various sources such as the scientific literature and/or from results of any other known evaluations of the intervention if it has been piloted/trialled in your area or by someone else in another area. In some cases, you may have access to both types of evidence, but in others you may be limited to one only. It is important that you read and consider both if available.

In this section, the target population is defined as the group of people the intervention is intended for. In some cases, your target population can be very specific, for example, those with a certain health condition/risk factor. In others, the target population can be broad, for example all those within a specific geographical area. It is important that a target population is defined as it will have an impact on future monitoring and evaluation activities.

Domain A4: Evidence of effectiveness	
Level of evidence available	
A4.1 What is the strength of evidence of effectiveness for the intervention in addressing the problem described in Domain A1 and A2, based on literature?<br><br>National Health and Medical Research Council evidence levels<br>I – Systematic review of level II evidence demonstrating benefit<br>II – RCT or Cluster RCT demonstrating benefit<br>III – 1 A pseudo-randomised controlled trial demonstrating benefit<br>III – 2 Comparative study with no concurrent controls (non-randomised experimental trial, cohort study, case-control study, interrupted time series with a control) demonstrating benefit<br>III – 3 Comparative study without concurrent controls (historical control study, two or more single arm study, interrupted time series analysis without a control group) demonstrating benefit<br>IV – Case series with either post-test or pre-test/post-test outcomes	Systematic review of level II evidence demonstrating benefit<br>A systematic review and meta-analysis by Kettle and colleagues (2022), found that physical activity intervention delivered or prompted by health professionals in primary care settings led to an increase in moderate-vigorous physical activity participation, and increased the likelihood of patients meeting the moderate-vigorous physical activity guidelines. It should be noted that while this review included studies in primary care settings, our study will primarily include clinical teams in hospital settings.<br>A systematic review and meta-analysis by Fontaine and colleagues (2024) explored the effect of implementation strategies on the practice of nurses and resulting patients outcomes. Included studies were conducted in hospital and community-based settings. The review found that group and individual education, reminders, patient-mediated interventions and involvement of opinion leaders had a positive impact on clinical practice outcomes. They also found that the inclusion of group and individual clinical education may lead to slight
A4.2 What was the size of the intervention effect (if known)?<br>(Mean difference, relative risk, odds ratio, hazard ratio, sensitivity, specificity and statistical significance)<br><br>Note: It is important to know that intervention effects generally decline from controlled setting to implementation at scale in the real world	Improvement of 14 min/week in MVPA (95% CI 4.2 to 24.6 min, p=0.006)<br>Increased odds of patients meeting guidelines for MVPA by 33% (05% CI 1.17 to 1.5, p<0.001)
A4.3 Describe core intervention components (as described in Domain A2) that contribute to intervention effectiveness (if known)	The review found that a greater number of contacts, and interaction with a primary health care professional and other interventionist, significantly increased self-reported MVPA.
A4.4 Is the effect size of the intervention meaningful from a population health policy perspective?	A 33% increased odds of patient meeting MVPA guidelines will contribute to the Australian govenment's target of reducing physcial inactivity by 15% by 2030. A

Domain A4: Evidence of effectiveness	
Note: A statistically significant difference, though a good start, is not necessarily a difference of policy/clinical significance. Intervention effects of policy/clinical significance are meaningful changes on an individual or group that, if scaled up, can make a substantial improvement to the outcome of interest	
A4.5 Did the intervention have differential effects on the target population?<br>Note: Differences in effectiveness amongst target populations/settings	This was not reported.
A4.6 Did the intervention have any known unintended consequences and/or adverse outcomes that were reported (in the literature or elsewhere)?<br>Note: Unintended consequences can be positive or negative	A systematic review and meta-analysis (Oloo et al., 2020) found no evidence of an increased risk of adverse events.
A4.7 Is there evidence that the intervention has a relative advantage over existing interventions to address the same problem?	Studies included in the systematic review (Kettle et al., 2020) that did not cross the line of no effect compared physical activity promotion interventions to brief advice (Pinto 2005), usual care (Elley 2003) [illegible]
A4.8 Has the intervention been implemented at a<br>(a) larger scale (either in literature or elsewhere) and/or<br>(b) other delivery settings (from original intention)?<br>If Yes to either, was it found to be effective?<br>Describe the results	The Green Prescription Program holds similarities to the community referrals and physical activity linkage element of our program, whereby a healthcare professionals refers a patient to a community-based provider to facilitate physical activity behaviour change (with support being provided through PA prescription, assistance with overcoming barriers to PA, team-based structure for [illegible]

3.5.1 Readiness assessment Domain A4: Evidence of effectiveness

Question	Not applicable	Not at all	To a small extent	Somewhat	To a large extent
**5.** Is there compelling evidence (from the literature or elsewhere) to indicate that the intervention is effective in addressing the problem in the target population?	N/A	0	1	2	3
**Total score for Domain A4**				2	

3.6 DOMAIN A5: INTERVENTION COSTS AND BENEFITS

This domain considers the known costs of the intervention delivery as well as any quantifiable benefits. Economic evaluation is dependent on information on the costs and benefits of programs. Methods include cost effectiveness analysis, cost benefit analysis, cost utility analysis, etc (4). In some circumstances, intervention costs may not be well known, but it is preferable that some indication of costs be gathered so that more informed consideration of scalability can be made.

Domain A5: Intervention costs and benefits	
Level of evidence available	
A5.1 What were the intervention costs reported (if available)?<br>Consider costs associated with start-up (e.g. building infrastructure, conducting training), costs associated with ongoing delivery as well as cost per participant or cost per unit of outcome	The reported cost of a physical activity counselling intervention in general practices in New Zealand found that the cost per participant was $NZ170 (2001) (Dalziel et al., 2005). This included costs of program set-up and co-ordination, regional sports trust patient support and general practice advice and follow-up.
A5.2 Was there any evidence of benefit outweighing the costs?<br>Describe any evidence that the benefits of the program outweighed the costs<br>Potential measures may include: incremental cost-effectiveness ratio, cost-benefit analysis, cost per QALY etc	A modelled cost-effectiveness analysis of the Victorian Active Script program found ICERs of : $69 per patient to become more active in the short term; $138 per patient to accrue a health benefit; $3647 per DALY saved; $48 924 per premature death averted. Dalziel and colleagues (2005) found an ICER of $NZ2 053 per QALY gained (2001) from a health system

3.6.1 Readiness assessment Domain A5: Intervention costs and benefits

Question	Not applicable	Not at all	To a small extent	Somewhat	To a large extent
**6.** Is there evidence that the benefits of the intervention exceeded the costs?	N/A	0	1	2	3
**Total score for Domain A5**			2		

4 PART B: INTERVENTION IMPLEMENTATION PLANNING

Part B considers the implementation of the intervention (as implemented currently or in the literature) as well as the proposed implementation for scale up.. This section covers four domains: 1) fidelity and adaptation; 2) reach and acceptability; 3) delivery settings and workforce, and 4) implementation infrastructure. As noted previously, while there may be some overlap with the information sought here with information required for future scale up implementation planning, completing this tool does not negate the need for a more detailed implementation scale up plan, if scale up is found to be warranted. The questions in Part B are designed to promote early thinking about potential implementation needs and strategies that would contribute to its potential for scalability.

4.1 DOMAIN B1: FIDELITY AND ADAPTATION

This domain considers whether there are any proposed changes to the intervention required for scale up. For example, if the original intervention (described in Domain A2) required the delivery of 10 separate elements and only 8 elements are to be delivered in the scale up, record this. Any known impacts of these changes should also be noted.

Domain B1: Fidelity and adaption	
Considerations for scale up – Consider what might change from the current situation if the intervention is to be scaled up	
B1.1: Will there be any changes and/or adaptations made to the intervention from what was described in Domain A2 if the intervention is scaled up?<br>(Yes, No, Unsure). If Yes, please indicate what those changes will be. Note: Adaptions to intervention components may have positive or negative impacts on intervention effectiveness	Changes may be made to the intervention depending on acceptability to health professionals in clinical teams. While it is expected that health professionals will assess their patients for current physical activity participation, competing clinical demands may limit their capacity to conduct the other components of the 5A's model. Health professionals may need to refer patients on to [illegible]
B1.2: Are those changes and/or adaptations likely to have any impact on the intended outcomes of the intervention as described in Domain A2?<br>(Yes, No, Unsure). If Yes, please indicate what those changes/impacts will be	No - tailoring of implementation strategies is directed towards optimising physical activity promotion practices within clinical teams based on their identified barriers and needs.
B1.3: How will intervention fidelity be monitored and maintained?	In the scaled-up version of the intervention, audits of clinical notes can be used as a pragmatic way to monitor fidelity.

4.1.1 Readiness assessment Domain B1: Fidelity and adaptation

Question	Not applicable	Not at all	To a small extent	Somewhat	To a large extent
**7.** Will the core components of the scaled up intervention be consistent with what was previously shown to be effective?	N/A	0	1	2	3
**8.** If the core components of intervention are to be changed/adapted from its original form during scale up, will the impact of the changes/adaptations likely be favourable?	N/A	0	1	2	3
**9.** Can program fidelity be monitored and/or maintained if implemented at scale?	N/A	0	1	2	3
**Total score for Domain B1**			7		

4.2 DOMAIN B2: REACH AND ACCEPTABILITY

This domain considers the reach and acceptability of the intervention for the target population.

Domain B2: Reach and acceptability	
**Previous/current situation**	
B2.1 Describe the target population for the intervention<br>Describe who was targeted in the literature or in pilot program	- Intervention: patients aged 5 years or older seeking healthcare in hospital and community-based settings who do
B2.2 How were the target population identified and recruited?<br>Describe how the target population(s) were identified and/or recruited in the literature or pilot program, e.g. what recruitment strategies were used	The recruitment of health professionals involved the research team approaching clinical teams and delivering a presentation on the background and elements of the implementation support program.<br>Health professionals within recruited clinical teams identified
B2.3 What was the level of participation and/or completion rate in the target population?<br>Describe the level of participation and/or rate of completion of the target population in the literature or pilot program	An Australia study by Zhu and colleagues (2021) investigating the physical activity promotion behaviours of physiotherapists in hospital settings found that 70% of physiotherapists [illegible]
B2.4 Was the intervention acceptable to the target population?<br>Was there any evidence (from literature or pilot program) to suggest that the intervention was acceptable to the intervention population?	A systematic review investigating the perceptions of health professionals on physical activity promotion found that the majority of health professionals agreed that physical activity promotion was important for their patients (Albert et al., [illegible]
**Considerations for scale up - Consider what might change from the current situation if the intervention is to be scaled up**	
B2.5 Describe the target population for the intervention at scale	- Intervention: patients aged 5 years or older seeking healthcare in hospital and community-based settings who do
B2.6 How will the intended target group be identified and recruited at scale?	Advertisements for implementation support could be released through LHD and hospital channels (e.g. newsletters), and
B2.7 Have there been any projections/estimations developed for scale up, to consider:<br>• Likely level of participation and/or completion rates of the target population<br>• Likely required timeframe required to achieve desired level of participation and/or reach?<br>If yes, what are they and how likely are they to be achieved within available resources?	The Active Script Programme was established in Victoria in 1999 to increase the number of Victorian GPs delivering physical activity advice to their patients. (Sims et al., 2004) All 31 Divisions of General Practice (DGP) were invited to submit expressions of interest to the program, of which 16 DGPs expressed interest. Around 40% of all Victorian GPs were involved in the program.
B2.8 Are there any foreseeable facilitators and/or barriers for reaching the target populations as part of the scale up process?<br>Facilitators or barriers in this case can be in terms of process, persons, practices, policies, budget	Both quantitative and qualitative research informing the development of the implementation strategies for PROMOTE identified a number of potential facilitators and barriers (Zhu 2021; Purcell 2023; West 2021).<br>[illegible]

4.2.1 Readiness assessment Domain B2: Reach and acceptability

Question	Not applicable	Not at all	To a small extent	Somewhat	To a large extent
**10.** Does the intervention have the potential to reach the intended target population at scale?	N/A	0	1	2	3
**11.** Is the intervention likely to be acceptable to the target population?	N/A	0	1	2	3
**Total score for Domain B2**	6				

4.3 DOMAIN B3: DELIVERY SETTING AND WORKFORCE

This domain considers the setting within which the intervention is delivered as well as the delivery workforce. In this domain, we refer to the delivery setting as the 'setting' in which the intervention is to be implemented, for example, schools, canteens, community, child care centres, hospitals. The delivery organisation, on the other hand, refers to the individual organisations that will implement the intervention. Delivery organisations may be newly created for the purpose of scaling up or they may already exist. Finally, the delivery workforce refers to those directly involved in delivering or administering the intervention to the target population.

Domain B3: Delivery setting and workforce	
Current situation	
B3.1 Describe the delivery setting/s where the intervention has been delivered<br>Describe where the intervention has been implemented (in pilot or literature). Delivery setting in this case means the context in which the intervention has been delivered e.g. schools, school canteens, sexual health clinics, community health centres, early childhood settings	The implementation support and physical activity promotion intervention is being delivered in the hospital setting as well as community-based private clinics.
B3.2 Describe the delivery workforce required for the administering the program/ intervention<br>Who were they and what did they do?	Research staff (4 team members) are delivering implementation support to clinical teams. This involves meeting with them to discuss strategies for PA promotion as
B3.3 Was the intervention deemed acceptable to the delivery workforce?<br>For example, was there any feedback from the delivery workforce in relation to the intervention? Consider feedback from referrers to/from the intervention as well	Early feedback from clinical teams has suggested the model of implementation support has been beneficial and they have been able to apply the knowledge and skills gained to promoting physical activity to their patients.
B3.4 How was the delivery workforce supported to deliver the intervention implementation?<br>For example, were resources provided to assist with implementation, how much time was required to assist with implementation?	Implementation strategies were delivered by the research team and tailored to each clinical team. With the first few early implementation teams, the research team has on average spent 30-60 min preparing, meeting and following up for clinical teams per occasion. Implementation strategies
B3.5 Were there any facilitators and/or barriers identified in the delivery setting when the intervention was implemented?<br>If yes, what were they?	Barriers:<br>- competing clinical demands - high volume of patients resulting in a lack of time available to engage with resources provided
Considerations for scale up - Consider what might change from the current situation if the intervention is to be scaled up	
B3.6 Will the intervention be implemented in the same settings at scale?<br>Yes, No, Unsure. If no, in which other settings will the intervention be implemented?	Yes
B3.7 Who will deliver the intervention at scale?<br>For example, will the same delivery workforce be used? Will the same referrers be involved at scale?	If found to be cost-effective, a case could be presented to local health districts to support research staff to provide implementation support initially, with the aim of developing
B3.8 Is the intervention likely to be acceptable to the delivery workforce involved if implemented at scale?	Early feedback suggests the implementation support and physical activity promotion is likely to be acceptable when
B3.9 Does the intervention require a small or a large departure from current practices and cultures of delivery organisations and workforce?	Small departure - it is expected that training and education can be provided to clinical teams as part of their professional development/team meeting time, and that physical activity

Domain B3: Delivery setting and workforce	
Note: Consider the impact the implementation of the intervention will have on current practices and cultures of the organisation and whether those impacts will be well received	
B3.10 Have there been any projections/estimations developed for scale up, to consider:<br>• Likely level of adoption/uptake rates of delivery organisations<br>• Likely required timeframe required to achieve desired level of adoption/uptake by delivery organisations<br>• Likely required timeframe to achieve the desired levels of resourcing/recruitment of the delivery workforce<br>If yes, what are they and how likely are they to be achieved within available resources?	As part of the Victorian Active Script program, 85% of GPs (n=299) advised their inactive patients to be more active, with 53% reporting they routinely assessed activity levels of new patients. Patient feedback suggested the majority of GPs did adopt the delivery of physical activity promotion, although the sample of patients surveyed was small (52 of 54 participants).<br>Recruitment of the delivery workforce in scale up of the PROMOTE-PA program will require additional financial resources.
B3.11 Are there similar programs/interventions already in place in the proposed delivery setting that might facilitate or hinder scale up?<br>Does the intervention duplicate other services or interventions already in place or link with or leverage existing settings and/or services?	Many clinical teams or health professionals within clinical teams are currently delivering some level of physical activity promotion, however their effectiveness in improving patient physical activity levels varies. This may serve as a facilitator (indicating positive attitudes towards the importance of [illegible]
B3.12 Are there any foreseeable facilitators and/or barriers for the delivery settings as part of the scale up process?<br>Facilitators or barriers in this case can be in terms of process, people, practices, policies, budget	Facilitators:<br>- Attitudinal: enthusiasm of staff who see the value of PA promotion<br>- Feedback on patient PA levels and the importance of their

4.3.1 Readiness assessment Domain B3: Delivery setting and workforce

Question	Not applicable	Not at all	To a small extent	Somewhat	To a large extent
**12.** Is the delivery setting(s) selected to deliver the program at scale consistent with that used in previous studies?	N/A	0	1	2	3
**13.** Is the delivery workforce selected to deliver the program at scale consistent with that used in previous studies?	N/A	0	1	2	3
**14.** Is the intervention likely to be acceptable to the delivery workforce involved in its delivery at scale?	N/A	0	1	2	3
**15.** If the intervention requires integration into existing organisational or community structures, how likely is it to be feasible?	N/A	0	1	2	3
**Total score for Domain B3**	9				

4.4 DOMAIN B4: IMPLEMENTATION INFRASTRUCTURE

This domain requires consideration of the potential implementation infrastructure required for scale up. As before, some of the answers to these questions may be known or could be extrapolated given known information. Implementation infrastructure in this case includes the organisational and workforce support systems required for implementation at scale including training, accreditation processes, competency frameworks, information and performance monitoring systems. Implementation support team in this instance refers to the additional human resources required to assist in the implementation at scale. Their roles may include, but are not limited to, providing assistance to delivery settings and workforce, managing or providing oversight of the scale up process, training and providing advice.

Domain B4: Implementation infrastructure	
Current situation	
B4.1 Describe the infrastructure requirements for the delivery of the program/ intervention<br>i.e. classrooms, clinic facilities, sporting fields, community centres, IT equipment, etc	- Access to electronic devices and internet to access online resources hub, allow for implementation support to be provided online as needed<br>- Research team providing implementation support to clinical
B4.2 Describe the operational requirements for delivery of the intervention<br>i.e. training, education, monitoring and feedback systems, accreditation processes etc	- Research staff skilled in delivering implementation support to clinical teams<br>- Health professionals with adequate skills, knowledge and [illegible]
B4.3 Were there facilitators and/or barriers to the creation and maintenance of implementation infrastructure?	Facilitators:<br>- a highly skilled research team with diverse skills and experience across all aspects needed for implementation eg interpersonal
Considerations for scale up - Consider what might change from the current situation if the intervention is to be scaled up	
B4.4 Have there been any projections/estimations made for scale up, to consider:<br>• Likely implementation infrastructure required<br>• Likely resources and timeframe required to build or procure the implementation infrastructure?<br>If yes, what are they and how likely are they to be achieved within available resources?	- Skilled staff and costs available for website maintenance (will be reliant on additional funding for scale-up)<br>- Availability of electronic devices and internet access to access online resources hub and allow for implementation support to be provided online as needed (likely to be readily available to clinical teams as part of the hospital/clinic environment)<br>- Potential development of a EMR-integrated physical activity [illegible]
B4.5 Will implementation at scale require the creation of an implementation support team?<br>If yes, could they be created within proposed resources?	The existing implementation support team possess the skills to deliver implementation support at scale. However, to deliver this within an expanded set of healthcare settings, additional funding will need to be acquired.
B4.6 Are there any foreseeable facilitators and/or barriers to building implementation infrastructure as part of the scale up process?<br>Facilitators or barriers in this case can be in terms of acceptability to workforce, changes to practice, workload, etc	Barriers:<br>- acceptability to workforce- high clinical workload impacting receipt of and engagement with implementation support<br>- staff changes, staff leave<br>- Differing attitudes of health professionals towards the importance/promotion of PA

4.4.1 Readiness assessment Domain B4: Implementation infrastructure

Question	Not applicable	Not at all	To a small extent	Somewhat	To a large extent
**16.** Are the implementation infrastructure requirements of the intervention/program feasible for scale up?	N/A	0	1	2	3
**Total score for Domain B4**	2				

4.5 DOMAIN B5: SUSTAINABILITY

The purpose of this domain is to consider the longer-term outcomes of the scale up, and how, once scaled up, the intervention could become sustainable over the medium to longer term. Some of these questions will be difficult to answer or, in some cases, impossible. However, they are listed to promote thinking and to facilitate planning, which may increase the likelihood of future success. It is worth noting that 'sustainability' is context dependent and it will be necessary to consider your context when determining what timeframe would be appropriate for the intervention to be considered sustainable or how best to define what sustainability means.

Domain B5: Sustainability of the intervention	
B5.1 What level of integration into existing service delivery settings or organisations will the intervention require if scaled up?<br>Also consider whether the level of integration is feasible or sustainable	It is expected that implementation support could be delivered as part of teams' professional development or clinical meeting time. Delivery of physical activity promotion to patients would take place during routine patient consultations.
B5.2 If the intervention is implemented at scale, will it require a large commitment of funds (initial or ongoing)?<br>If yes to either or both, consider if an internal funding model such as co-payment schemes or sourcing from other agencies is possible, or whether it would be feasible to implement a self-funding model to pay for parts or all of the intervention through co-payments from individuals or organisations	If implemented at scale, it is likely that initial funding will be required for research staff to deliver implementation support to the new clinical teams. As the skills and knowledge of health professionals/clinical teams within different healthcare organisations are developed, it is expected that learnings will be shared across clinical teams within organisations (in a train-the-trainer model) to promote the practice of physical activity promotion to patients as part of routine clinical practice. [illegible]
B5.3 Is the proposed delivery workforce required for implementation at scale sustainable (e.g. financially and/or in terms of supply)?<br>Is there an alternative delivery workforce that can deliver the implementation at scale, e.g. using fitness leaders to deliver exercise classes instead of physiotherapists	Research staff required to deliver implementation support would be available to deliver this support/train other teams members as required. However, this would require additional funding to be sought. There is potential for implementation support to be delivered by a team leader/clinical champion in a train-the-trainer [illegible]

4.5.1 Readiness assessment Domain B5: Sustainability

Question	Not applicable	Not at all	To a small extent	Somewhat	To a large extent
**17.** Is the level of integration of the intervention into delivery settings required for implementation at scale sustainable?	N/A	0	1	2	3
**18.** Is the level of resourcing required to implement the intervention at scale sustainable?	N/A	0	1	2	3
**19.** Is the delivery workforce selected for implementation at scale sustainable?	N/A	0	1	2	3
**Total score for Domain B5**	6				

5 PART C: SUMMARY OF SCALABILITY ASSESSMENT

In this section, provide a brief summary based on the information gathered from the above sections.

Domains	Summary	Overall score
Domain A1: The problem	Almost one in two Australians do not meet the physical activity guidelines. Physcial inactivity is	3
Domain A2: The intervention	Implementation support for physical activity promotion will be provided by the research team	2
Domain A3: Strategic/political context	The Australian government has hihghlighted the important of preventive healthcare and recognises physical acitivity as a key part of this, with a goal to reduce physical inactivity by 15%	6
Domain A4: Evidence of effectiveness	A systematic review and meta-analysis has found that physical activity intervention delivered or	2
Domain A5: Intervention costs and benefits	Economic evaluations of physical activity promotion interventions previously conducted in Australia and New Zealand suggest that this intervention may be a cost-effective option in	2
Domain B1: Fidelity and adaptation	The core components of implementation support and intervention delivery are likely to be	7
Domain B2: Reach and acceptability	Previous research suggests that the intervention is acceptable to health professionals, and early	6
Domain B3: Delivery settings and workforce	hospitals and private practices. Research staff will deliver implementation support to health professionals, who will	9
Domain B4: Implementation infrastructure	Key implementation infrastructure required relate to techonology and skilled staff. Additional financial resources will be required for scale up	2
Domain B5: Sustainability	It is expected that the delivey of physical activity promotion will become embedded within	6

[INSERT VISUAL REPRESENTATION HERE]

Recommendation

- [ ] Merits scale up
- [x] Promising, but further information/planning is warranted
- [ ] Does not merit scale up

ATTACHMENT A: REPORT TEMPLATE

Intervention Scalability Assessment Tool Summary Assessment

PART A: SETTING THE SCENE

1. **The problem (200 words)**

Describe the problem, who does it affect and current practice to address the issues

2. **The intervention (200 words)**

Describe the intervention, its components and its intended outcome/s

3. **The strategic and political context (200 words)**

Describe how the intervention fits with the strategic context of the funder e.g. national and state priorities

4. **The evidence of effectiveness (200 words)**

Provide evidence of effectiveness of the intervention, differential effects and any unintended consequences

5. **Intervention costs and benefits (100 words)**

Provide evidence of benefits outweighing the costs

PART B: IMPLEMENTATION PLANNING

6. **Implementation planning considerations (600 words)**

Provide evidence of the potential implementation scale up considerations including:

How the intervention may or may not be adapted and how fidelity will be maintained and monitored; the likely reach and acceptability of the intervention to the target population; the likely acceptability to the proposed delivery setting and/or workforce; the likely implementation infrastructure required for scale up.

7. **Sustainability considerations (200 words)**

If considered, describe briefly if and how sustainability might be achieved over the medium to long term, particularly with regards to operational, financial and/or delivery workforce sustainability.

References

1. World Health Organization. Nine Steps for Developing a Scaling-Up Strategy. Geneva: WHO ExpandNet; 2010.
2. Milat AJ, King L, Bauman AE, Redman S. The concept of scalability: increasing the scale and potential adoption of health promotion interventions into policy and practice. Health Promotion International. 2013;28(3):285-98.
3. Milat AJ, Newson R, L. K. Increasing the scale of population health interventions: A guide. Sydney: NSW Ministry of Health, Division PaPH; 2014.
4. Centre for Epidemiology and Evidence . Commissioning Economic Evaluations: A Guide. Population and Public Health Division NSW Ministry of Health ; 2017.
